# Two complete mitochondrial genomes of the family Paradoxosomatidae (Diplopoda, Polydesmida) with phylogenetic implications

**DOI:** 10.3897/zookeys.1270.175945

**Published:** 2026-02-26

**Authors:** Ming Gao, Gaoji Zhang, Yingzhu Li, Xiaxi Jia, Yuting Ding, Hongyi Liu

**Affiliations:** 1 The Co-Innovation Center for Sustainable Forestry in Southern China, College of Life Sciences, Nanjing Forestry University, Nanjing 210037, China College of Life Sciences, Nanjing Forestry University Nanjing China

**Keywords:** Diplopoda, mitochondrial genomes, Paradoxosomatidae, phylogeny

## Abstract

Millipedes (class Diplopoda) are vital soil invertebrates that play key roles in litter decomposition and nutrient cycling. However, their phylogenetic relationships remain poorly resolved due to limited genomic resources. In this study, we sequenced and characterized the complete mitochondrial genomes (mitogenomes) of two paradoxosomatid millipedes, *Oxidus gracilis* (15,034 bp) and *Kronopolites swinhoei* (15,277 bp). Both mitogenomes contain the typical set of 37 genes, all located on the minor strand (N-strand), and display high AT content. The conserved gene arrangement observed here may represent a molecular synapomorphy for this taxonomic group. Analysis of codon usage revealed that start codons includ ATN (ATA/ATG/ATT), TTG, and GTG, while stop codons consisted of TAN (TAA/TAG/TAT) and an incomplete single T. Relative synonymous codon usage (RSCU) analysis indicated that Leu2, Val, and Gly were the most frequently used codon families, whereas Gln, Cys, and Lys were the least utilized. The tRNA genes formed two distinct clusters, and the rRNAs were flanked by tRNA-Val. The D-loop region was located in a similar position in both species. Phylogenetic reconstruction based on 13 protein-coding genes from 34 diplopod species, using both Bayesian inference and maximum likelihood methods, strongly supported the interordinal relationships among Julida, Spirostreptida, and Spirobolida, and placed *O. gracilis* and *K. swinhoei* in distinct clades. Our findings provide valuable mitogenomic data and new phylogenetic insights into Diplopoda, underscoring the importance of expanded taxonomic sampling to further elucidate evolutionary relationships within this ecologically significant group.

## Introduction

Soil invertebrates are indispensable to ecosystem functioning, driving fundamental processes such as litter decomposition, soil formation, and nutrient cycling. They enhance the availability of key nutrients (C, N, P, K) and improve soil physical and microbial properties (Jegede et al. 2019; Wu et al. 2023; Wu et al. 2025). Millipedes, as key soil invertebrates, play a critical role in decomposing litter (particularly fallen leaves) and maintaining soil fertility (Culliney 2013). Millipedes are classified under the class of Diplopoda, with over 18,000 described species (Golovatch and Liu 2020). The majority of millipede species are currently identified primarily through morphological approaches (Mesibov 2019). However, molecular and biogeographic evidence indicates the existence of a considerable number of undescribed species, with previous estimates ranging from 15,000 to 80,000 (Sierwald and Bond 2007; Shear 2011). Morphological identification for millipedes, while historically predominant, exhibits notable limitations due to the group’s high diversity and the prevalence of homoplastic traits shaped by environmental adaptations (Liu et al. 2017).

Traditional identification methods face some challenges in identification (Engh et al. 2003). Mitochondrial genome (mitogenome) exhibits several distinctive characteristics, including strict maternal inheritance, rapid evolutionary rate, and an absence of genetic recombination (Tuli et al. 2022). Their mitochondrial DNA is characterized by a closed, circular, double-stranded configuration and undergoes semi-conservative replication (Scarpulla 2008). The mitogenome is typically composed of 37 coding elements, comprising 13 protein-coding genes (PCGs), 22 transfer RNA genes (tRNAs), 2 ribosomal RNA genes (rRNAs), and a D-loop region (Xu et al. 2024, 2025). As an ideal genetic marker, it provides a novel, even completely new, perspective for species identification, evolutionary analysis, and phylogenetic inference. Extensive reorganization of both tRNAs and PCGs was found among and within the subphyla Chelicerata, Myriapoda, and Crustacea using mitogenome (Sterling-Montealegre and Prada 2023). The mitogenomes of four millipede species were sequenced to investigate evolutionary relationships among diplopod taxa and characterize their genomic features (Li et al. 2025). Currently, complete mitogenome sequences are available for fewer than 50 millipede species, highlighting a significant gap in genomic understanding of millipedes (Li et al. 2025). A robust and comprehensive phylogenetic analysis of the class Diplopoda is contingent upon the acquisition of a substantial number of complete mitogenomes from its diverse subordinate species (Golovatch and Liu 2020).

The phylogenetic position of the family Paradoxosomatidae within the order Polydesmida remains underexplored and poorly resolved (Yang et al. 2018; Zhang et al. 2024b). Due to the scarcity of robust molecular phylogenetic frameworks for this family, the evolutionary relationships between Paradoxosomatidae and other polydesmidan lineages are still unclear. To address this gap, we sequenced and characterized the complete mitochondrial genomes of two paradoxosomatid species: *Oxidus gracilis* (Koch, 1847) and *Kronopolites swinhoei* (Pocock, 1895). Their mitogenomic analysis not only provides insights into gene rearrangement patterns within Paradoxosomatidae but also contributes to broader efforts in documenting the undersampled phylogenetic diversity of millipedes. We assembled and annotated the mitogenomes of *O. gracilis* and *K. swinhoei* and conducted a comprehensive analysis of key parameters, including genome size, nucleotide composition, codon usage, AT-skews, and GC-Skew. To resolve evolutionary relationships within Diplopoda, we employed Bayesian inference (BI) and maximum likelihood (ML) methods on these whole mitogenome data to reconstruct a robust phylogenetic framework. This study aims to provide new molecular evidence for characterizing millipede mitogenomes and to refine the phylogenetic relationships within millipedes group.

## Materials and methods

### Sample collection and identification

Specimens of *O. gracilis* and *K. swinhoei* were originally collected from two distinct localities in Nanjing, China: Mufu Mountain (32°07'N, 118°47'E) and Zijin Mountain (32°04'N,118°51'E). The identification of these millipedes was performed based on diagnostic morphological characteristics following established taxonomic descriptions (Iniesta et al. 2020; Xiong et al. 2025). Total genomic DNA was extracted using the DNAiso (Takara, Beijing, China) following the manufacturer’s protocol. All extracted DNA samples were archived at -80 °C in the Zoology Laboratory of Nanjing Forestry University for long-term preservation and future use.

### Next-Generation sequencing, mitochondrial genome assembly, and annotation

The sequencing procedure was conducted following established Illumina next-generation sequencing protocols. Genomic DNA was initially fragmented to approximately 350 bp using a Covaris ultrasonication system, followed by end repair, A-tailing, and ligation of full-length Illumina adapters for library construction. Sequencing was performed using a PE150 paired-end strategy. Raw sequencing data underwent stringent quality control through Fastp, with filtering criteria including: (1) removal of reads containing adapter sequences (>10 bp alignment); (2) exclusion of reads with > 10% undetermined bases, and (3) elimination of reads where > 50% of bases had Phred quality scores below 5. This process yielded high-quality data for subsequent bioinformatic analyses. All procedures described above were conducted by Novogene (Nanjing, China). Geneious Prime v2024.0.7 was employed to assemble the complete mitogenome through reference-guided assembly, utilizing the published genomes of *Abacion magnum* and *Nedyopus patrioticus* as reference templates. Assembly was performed under medium sensitivity/speed parameters, yielding a consensus sequence with a 99% base-call threshold. The circular structure of the assembled mitogenome was confirmed by manual inspection of the terminal region alignments. The two mitogenomes underwent preliminary examination using Seqman v7.1.0 (Jin and Sun 2018) and were subsequently annotated with the MITOS WebServer (Meng et al. 2019) to determine the precise genomic locations of tRNAs, rRNAs, the D-loop region, and all PCGs.

### Sequence analysis and phylogenetic analysis

Genomic visualization was performed by constructing a circular diagram with Proksee (Subbiah V.K. et al. 2024). Relative synonymous codon usage (RSCU) was conducted in MEGA11 (Kumar et al. 2018), and visualized with PhyloSuite v1.2.3 (Zhang et al. 2020). Nucleotide composition skewness was assessed based on established formulae: AT-skew = (A − T)/(A + T) and GC-skew = (G − C)/(G + C) (Perna and Kocher 1995).

The phylogenetic relationships among millipede groups were reconstructed based on the complete mitogenomes of 34 species, using *Cermatobius longicornis* (Takakuwa, 1939) as the outgroup (Table 1). The phylogenetic dataset was derived from the concatenated sequences of the complete suite of 13 PCGs of mitochondrial genes. Phylogenetic analyses for each dataset were conducted through both ML and BI using the integrated PhyloSuite platform v1.2.3 (Zhang et al. 2020; Xiang et al. 2023). Sequence alignment was performed using MAFFT v.7.313 (Rozewicki et al. 2019), and the best-fit substitution model was selected under the Bayesian Information Criterion (BIC) in ModelFinder (Kalyaanamoorthy et al. 2017). The selected models were GTR+F+R5 for ML and GTR+F+I+G4 for BI.. ML trees were reconstructed in IQ-TREE v2.2.0 (Nguyen et al. 2015) with 5,000 ultrafast bootstrap replicates (Minh et al. 2013) under the GTR+F+R5 model. BI was carried out in MrBayes v3.2.7a (Ronquist et al. 2012) with two independent Markov chain Monte Carlo (MCMC) runs of 2,000,000 generations each, sampling every 1000 steps. The first 25% of trees were discarded as burn-in after confirming convergence, as indicated by an average standard deviation of split frequencies (ASDSF) below 0.01.

**Table 1.** The mitogenomes used in phylogenetic analyses. Names in bold indicate the two Paradoxosomatidae species newly sequenced in this study.

Class	Order	Family	Species	Length (bp)	Accession
Diplopoda	Callipodida	Callipodidae	*Abacion magnum* Loomis, 1943	15,160	JX437062.1
	Platydesmida	Andrognathidae	*Brachycybe lecontii* Wood, 1864	15,115	JX437064.1
	Polydesmida	Xystodesmidae	*Appalachioria falcifera* Keeton, 1959	15,282	JX437063.1
			*Xystodesmus* sp. YD-2016 Cook, 1895	15,791	KU721886.1
		Paradoxosomatidae	*Asiomorpha coarctata* DeSaussure, 1860	15,644	KU721885.1
			***Kronopolites swinhoei* Pocock, 1895**	**15,277**	**PX619173**
			*Nedyopus patrioticus* Attems, 1898	15,814	OR755973.1
			*Nedyopus patrioticus unicolor* Carl, 1902	15,798	OR777861.1
			***Oxidus gracilis* C. L. Koch, 1847**	**15,034**	**PX603245**
		Polydesmidae	*Epanerchodus koreanus* Verhoeff, 1937	15,581	MT898420.1
	Spirobolida	Trigoniulidae	*Trigoniulus corallinus* Gervais, 1847	14,906	PQ459337
		Pachybolidae	*Litostrophus scaber* Verhoeff, 1938	15,081	OR139892.1
		Spirobolellidae	*Paraspirobolus lucifugus* Gervais, 1836	14,929	PQ625794
		Spirobolidae	*Narceus annularus* Rafinesque, 1820	14,868	AY055727.1
			*Spirobolus bungii* Keeton, 1960	14,879	MT767838.1
			*Spirobolus grahami* Keeton, 1960	14,875	OR038162.1
			*Spirobolus walkeri* Pocock, 1895	14,879	OR078377.1
	Julida	Nemasomatidae	*Antrokoreana gracilipes* Verhoeff, 1938	14,747	DQ344025.1
		Julidae	*Anaulaciulus koreanus* Verhoeff, 1937	14,916	KX096886.1
	Spirostreptida	Cambalopsidae	*Bilingulus sinicus* Zhang & Li, 1981	15,879	PQ568882
		Odontopygidae	*Prionopetalum kraepelini* Attems, 1896	15,114	MT394524.1
			*Chaleponcus netus* Enghoff, 2014	15,093	MT394513.1
		Harpagophoridae	*Agaricogonopus acrotrifoliolatus* Zhang, 1997	15,016	PQ602473
		Spirostreptidae	*Pseudotibiozus cerasopus* Attems, 1914	15,121	MT394506.1
			*Archispirostreptus gigas* Peters, 1985	15,177	MT394525.1
			*Macrolenostreptus oreste* Hoffman & Howell, 1996	15,367	MT394512.1
			*Tropostreptus severus* Enghoff, 2017	15,209	MT394517.1
			*Tropostreptus austerus* Attems, 1950	15,261	MT394523.1
			*Tropostreptus hamatus* Demange, 1977	15,156	MT394508.1
			*Tropostreptus kipunji* Enghoff, 2017	15,170	MT394503.1
			*Tropostreptus droides* Enghoff, 2017	15,172	MT394522.1
			*Tropostreptus sigmatospinus* Enghoff, 2017	15,176	MT394504.1
			*Tropostreptus microcephalus* Enghoff, 2017	15,169	MT394516.1
Chilopoda	Lithobiomorpha	Henicopidae	*Cermatobius longicornis* Takakuwa, 1939	16,833	KC155628.1

## Results and discussion

### Mitochondrial genome organization

**Figure 1.**
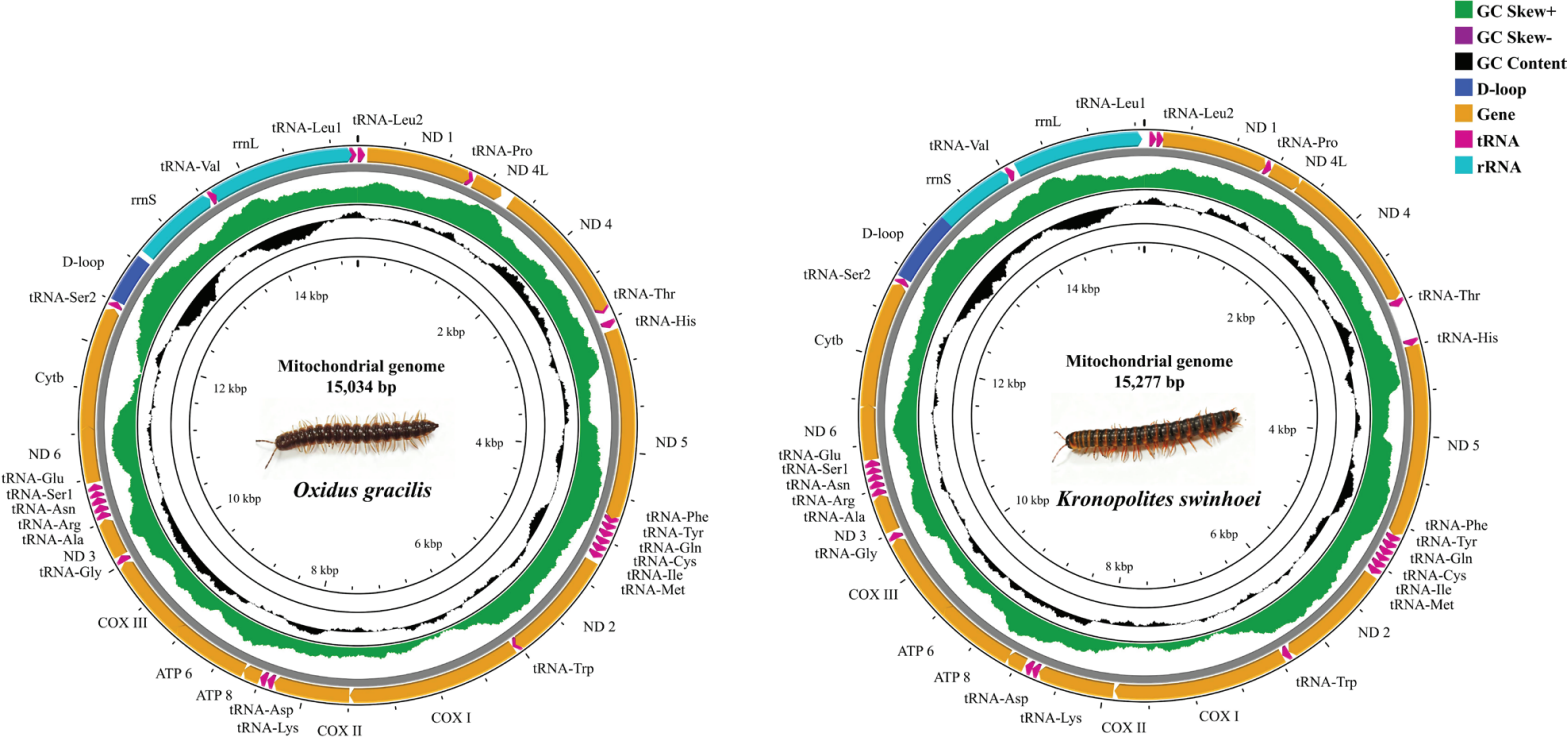
Mitochondrial genomes of *Oxidus gracilis* Koch, 1847 and *Kronopolites swinhoei* Pocock, 1895.

The complete mitogenomes of *O. gracilis* and *K. swinhoei* were characterized as circular double-stranded DNA molecules, with lengths of 15,034 bp and 15,277 bp, respectively (Fig. 1). Each assembly contained the complete set of 37 mitochondrial genes: 13 PCGs, 22 tRNAs, 2 rRNAs and a D-loop region. All genes in both mitogenomes were located on the minor strand (N-strand). Notably, this N-strand localization pattern is shared with other members of the family Paradoxosomatidae, including *Nedyopus patrioticus patrioticus* and *Nedyopus patrioticus unicolor* (Zhang et al. 2024b). This genomic arrangement was also observed across other polydesmidan lineages, suggesting that it may represent a conserved trait within the order (Dong et al. 2016; Joo et al. 2020). Gene overlaps were also identified in the mitogenomes of both millipede species. The longest intergenic overlap reached 47 bp between ND6 and Cytb in *O. gracilis*, whereas it extended to 38 bp between ATP6 and COX III in *K. swinhoei* (Table 2). Similar overlapping phenomena have been reported in other Diplopoda and soil invertebrates (Zhang et al. 2024b; Xu et al. 2025). Under evolutionary pressure toward genomic compactness, gene overlap represents an economically efficient strategy, likely reflecting selection for energy conservation and high efficiency in genetic material replication and maintenance (Doublet et al. 2015).

**Table 2.** General features of the mitogenomes of *O. gracilis* and *K. swinhoei*.

Gene	Location		Intergenic Nucleotides	Size (bp)
	From	To		
tRNA-Leu2	1 / 109	66 / 171	10 / -1	66 / 63
ND1	86 / 166	992 / 1104	19 / -6	907 / 939
tRNA-Pro	993 / 1098	1057 / 1160	0 / -7	65 / 63
ND4L	1064 / 1162	1339 / 1443	6 / 1	276 / 282
ND4	1414 / 1434	2674 / 2771	74 / -10	1261 / 1338
tRNA-Thr	2675 / 2772	2741 / 2838	0 / 0	67 / 67
tRNA-His	2814 / 3140	2882 / 3201	72 / 301	69 / 62
ND5	2885 / 3199	4583 / 4905	2 / -3	1699 / 1707
tRNA-Phe	4584 / 4905	4650 / 4970	0 / -1	67 / 66
tRNA-Tyr	4651 / 4970	4713 / 5036	0 / -1	63 / 67
tRNA-Gln	4716 / 5037	4781 / 5102	2 / 0	66 / 66
tRNA-Cys	4788 / 5104	4853 / 5164	6 / 1	66 / 61
tRNA-Ile	4855 / 5165	4920 / 5230	1 / 0	66 / 66
tRNA-Met	4922 / 5231	4985 / 5295	1 / 0	64 / 65
ND2	5016 / 5308	5994 / 6303	30 / 12	1009 / 996
tRNA-Trp	5995 / 6304	6057 / 6369	-30 / 0	63 / 66
COX I	6059 / 6371	7591 / 7903	1 / 1	1533 / 1533
COX II	7595 / 7913	8272 / 8590	3 / 9	678 / 678
tRNA-Lys	8273 / 8591	8336 / 8655	0 / 0	64 / 65
tRNA-Asp	8340 / 8655	8404 / 8720	3 / -1	65 / 66
ATP8	8405 / 8730	8563 / 8894	0 / 9	159 / 165
ATP6	8560 / 8888	9223 / 9592	-4 / -7	664 / 705
COX III	9224 / 9555	10009 / 10340	0 / -38	786 / 786
tRNA-Gly	10011 / 10340	10075 / 10402	1 / -1	65 / 63
ND3	10077 / 10418	10427 / 10753	1 / 15	351 / 336
tRNA-Ala	10436 / 10752	10497 / 10814	8 / -2	62 / 63
tRNA-Arg	10498 / 10818	10563 / 10882	0 / 3	66 / 65
tRNA-Asn	10567 / 10884	10632 / 10946	3 / 1	66 / 63
tRNA-Ser1	10633 / 10947	10690 / 11005	0 / 0	58 / 59
tRNA-Glu	10694 / 11007	10755 / 11070	3 / 1	62 / 64
ND6	10768 / 11087	11271 / 11560	12 / 16	504 / 474
Cytb	11225 / 11541	12343 / 12662	-47 / -20	1119 / 1122
tRNA-Ser2	12355 / 12663	12411 / 12719	11 / 0	57 / 57
D-loop	12412 / 12720	12848 / 13343	0 / 0	437 / 624
rrnS	12904 / 13344	13649 / 14025	55 / 0	746 / 682
tRNA-Val	13654 / 14023	13717 / 14087	4 / -3	64 / 65
rrnL	13698 / 14117	14983 / 15259	-20 / 29	1286 / 1143
tRNA-Leu1	14961 / 46	15024 / 107	-23 / 63	64 / 62

In general, both mitogenomes exhibited highly conserved organizational features. Nucleotide composition of *O. gracilis* was A: 25.22%, T: 40.80%, G: 23.76%, C: 9.63%, while that of *K. swinhoei* was A: 20.46%, T: 40.15%, G: 30.29%, C: 9.10%. A notably high T and low C content were observed as a shared characteristic in both species. Based on nucleotide composition analyses of Polydesmida species, the genomes exhibited a marked AT bias, with values ranging from 60.61% in *K. swinhoei* to 75.11% in *E. koreanus* Verhoeff, 1937, while the corresponding G and C contents were notably low (Table 3). High overall AT content observed in the studied millipedes may represent a molecular characteristic common to invertebrates (Gissi et al. 2008). This view was further supported by the detection of a similarly elevated AT content (77.8%) in another invertebrate, *Thrips hawaiiensis* (Wang et al. 2021). Interestingly, all AT-skew values were negative (-0.22~-0.37) and all GC-skew were positive (0.42~0.54). These skew patterns suggested strand-specific selection and mutational pressures collectively drive the high AT content in millipede mitogenomes (Gissi et al. 2008).

**Table 3.** Base compositions of the Polydesmida mitogenomes.

Species	Whole genome				PCGs		tRNAs		rRNAs		D-loop	
	Size (bp)	AT (%)	AT skew	GC skew	Size (bp)	AT (%)	Size (bp)	AT (%)	Size (bp)	AT%	Size (bp)	AT%
*O. gracilis*	15,034	66.02	-0.24	0.42	10,916	64.49	1415	68.34	2032	70.13	437	73.46
*K. swinhoei*	15,277	60.61	-0.32	0.54	11,061	59.47	1404	64.17	1825	63.89	624	70.19
*A. coarctata*	15,644	67.44	-0.26	0.43	11,021	66.11	1482	69.84	2016	69.35	954	75.68
*A. falcifera*	15,282	64.04	-0.37	0.44	11,009	63.12	1399	66.40	2025	68.74	880	60.57
*E. koreanus*	15,581	75.11	-0.26	0.45	10,965	73.89	1371	77.10	2082	79.06	411	69.34
*N. patrioticus*	15,814	68.15	-0.26	0.46	10,904	66.99	1203	67.66	2086	72.63	1168	67.72
*X.* sp.	15,791	67.01	-0.22	0.47	11,030	65.62	1363	72.05	2007	69.21	1032	70.06

### PCGSs and codon usage

The combined length of PCGs was 10,916 bp in *O. gracilis* and 11,061 bp in *K. swinhoei*, accounting for 72.61% and 72.40% of their respective complete mitogenomes. Among these PCGs, ND5 was the largest and ATP8 the smallest in both species (Table 2). This pattern is also observed in other diplopods, including *Agaricogonopus acrotrifoliolatus*, *Bilingulus sinicus*, *Paraspirobolus lucius*, and *Trigoniulus corallinus* (Li et al. 2025). As shown in Fig. 1, the arrangement of all PCGs was conserved between the two species: ND1, ND4L, ND4, ND5, ND2, COX I, COX II, ATP8, ATP6, COX III, ND3 and Cytb. Due to the structural constraints imposed by the circular construction of mitogenomes, an identical arrangement of PCGs is maintained and observed in *N. patrioticus* (Zhang et al. 2024b). Notably, as *N. patrioticus*, *O. gracilis*, and *K. swinhoei* all belong to the family Paradoxosomatidae, this shared gene arrangement may serve as a diagnostic molecular information for the family.

**Figure 2.**
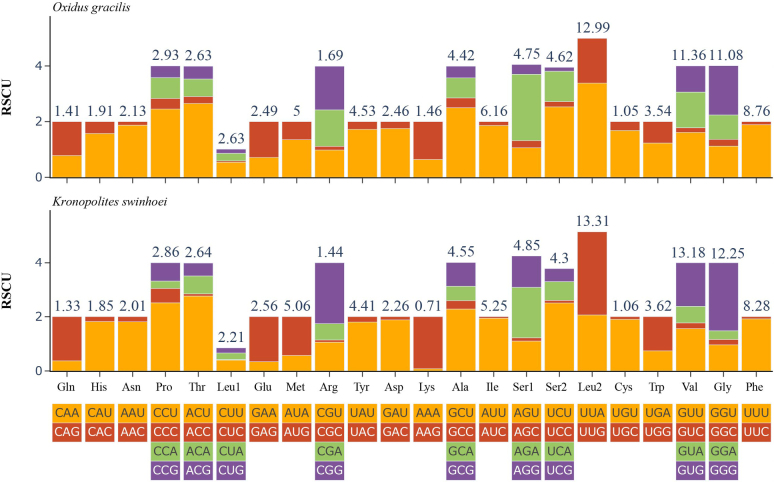
RSCU values of two species of millipedes. Termination codon is not included.

Analysis of codon usage within Polydesmida revealed that most PCGs were found to initiate with an ATN (ATA/T/G) as start codon, while a minority utilized TTG or GTG as alternative start codons (Table 4). *Spirobolus bungii* was also observed to follow this pattern of start and stop codon usage (Xu et al. 2022). In *O. gracilis*, eight PCGs (ATP8, COX I, COX II, COX III, Cytb, ND1, ND3, ND5) started with ATG; three (ATP8, ND4L, ND6) with ATA; and two (ND2, ND4) with ATT. In *K. swinhoei*, eight PCGs (ATP6, ATP8, COX I, COX II, COX III, Cytb, ND2, ND4L) used ATG; four used ATT; and only ND1 used TTG. The unusual start codon TTG in ND1 was also reported in *Asiomorpha coarctata* and *Xystodesmus* sp. (Dong et al. 2016). The majority of termination codons were typical TAN codons (TAA/TAG/TAT), with a single nucleotide T notably serving as a stop codon. This abbreviated termination signal was also documented in other soil invertebrates (Zhu et al. 2018; Cheng et al. 2021). It was hypothesized that such incomplete codons were post-transcriptionally modified into complete stop codons (TAA or TAG) through polyadenylation to enable proper translation termination (Xu et al. 2025). Furthermore, these incomplete stop codons may be located adjacent to tRNAs at the 3’ ends, a configuration considered to result from post-transcriptional polyadenylation of tRNA (Lin et al. 2025). The codon usage characteristics of *O. gracilis* and *K. swinhoei* were investigated through comparative analysis of RSCU (Fig. 2). As shown in Fig. 2, both species exhibited highly similar RSCU profiles. The three most frequently used codon families encoded Leu2, Val, and Gly, whereas Gln, Cys, and Lys represented the three least utilized codon families. Codons ending with A or U showed significantly higher usage frequencies than those ending with C or G, reflecting a pronounced AT bias at the third codon position. This pattern was also reported in *Litostrophus scaber* (Zhang et al. 2024a).

**Table 4.** Comparison of start and stop codons of mitochondrial PCGs in Polydesmida.

Gene	Start codon / stop codon						
	*O. gracilis*	*K. swinhoei*	*A. coarctata*	*A. falcifera*	*E. koreanus*	*N. patrioticus*	*X.* sp.
ATP6	ATA/T	ATG/TAG	ATG/T	GTG/T	GTG/T	ATA/TAA	ATG/TAA
ATP8	ATG/TAG	ATG/TAG	ATG/TAA	GTG/TAG	TTG/T	ATG/TAG	ATG/TAA
COX I	ATG/TAA	ATG/TAG	ATG/TAG	ATG/TAA	GTG/TAA	ATG/TAG	ATG/TAG
COX II	ATG/TAA	ATG/TAA	ATG/TAG	ATG/T	ATG/T	ATG/TAA	ATG/TAG
COX III	ATG/TAA	ATG/TAA	ATG/TAA	ATA/TAG	ATG/TAG	ATG/TAA	ATG/TAG
Cytb	ATG/TAA	ATG/TAG	ATG/TAA	ATG/TAG	ATG/TAT	ATG/TAG	ATG/TAA
ND1	ATG/T	TTG/TAG	TTG/T	GTG/T	ATG/TAG	ATA/TAT	TTG/T
ND2	ATT/T	ATG/TAG	TTG/TAA	TTG/T	GTG/TAA	ATT/TAG	TTG/TAA
ND3	ATG/TAG	ATT/TAG	GTG/TAA	TTG/T	ATA/TAA	ATT/TAG	GTG/TAG
ND4	ATT/T	ATT/TAG	GTG/TAG	ATG/TAG	ATT/TAA	ATG/TAG	GTG/TAA
ND4L	ATA/TAG	ATG/TAG	ATG/TAG	ATG/TAG	ATG/T	ATG/TAG	ATG/TAG
ND5	ATG/T	ATT/TAG	ATG/TAG	ATG/TAA	ATG/T	ATG/T	ATG/TAG
ND6	ATA/TAA	ATT/TAA	ATG/T	TTG/T	TTG/T	ATA/TAA	ATG/T

### tRNAs, rRNAs, and D-loop

The mitogenomes of both millipede species contained 22 tRNA genes. Analysis across Polydesmida members revealed total tRNA lengths ranging from 1203 bp to 1482 bp, with *O. gracilis* and *K. swinhoei* measuring 1415 bp and 1404 bp, respectively (Table 3). The AT content of these tRNAs varied between 64.17% and 77.10%, reflecting a significant AT bias consistent with the overall mitogenomic composition. Individual tRNA genes ranged from 57 bp to 69 bp in length (Table 2). Both *O. gracilis* and *K. swinhoei* shared conserved tRNA clusters, with identical gene arrangements observed in two distinct regions: the tRNA-Phe–Tyr–Gln–Cys–Ile–Met cluster and the tRNA-Ala–Arg–Asn–Ser1–Glu cluster. These specific tRNAs arrangement has also been reported in other members of Paradoxosomatidae (Zhang et al. 2024b).

Both mitogenomes contained rrnL and rrnS, separated by a tRNA-Val gene. This structural pattern, in which the two rRNAs are flanked by tRNA-Val gene, has also been reported in other Paradoxosomatidae species (Yang et al. 2018). As shown in Table 3, total rRNA length ranged from 1825 bp to 2082 bp across the examined Polydesmida. *O. gracilis* contained 2032 bp of rRNAs, while *K. swinhoei* had the minimal value at 1825 bp. These rRNAs sequences also exhibited significant AT bias, ranging from 63.89% to 79.06%, consistent with the overall base composition of the complete mitogenomes.

The D-loop region was identified between tRNA-Ser2 and rrnS in both *O. gracilis* and *K. swinhoei*. As summarized in Table 3, its length exhibited substantial variation across Polydesmida species, spanning from 411 bp to 1168 bp. A difference of 186 bp was observed specifically between *O. gracilis* and *K. swinhoei*. This divergence in length may reflect evolutionary dynamics shaping the D-loop region, analogous to those influencing PCGs and other mitogenomic components (Zhang et al. 2023). As the longest non-coding region in mitogenomes, the D-loop displayed characteristically high A-T content, ranging from 60.57% to 75.68% across the Polydesmida (Table 3), with values of 73.46% in *O. gracilis* and 70.19% in *K. swinhoei*. The AT-rich character of D-loop regions was consistent with previous reports on mitochondrial D-loop regions in other soil invertebrates (Liu et al. 2020).

### Phylogenetic analyses

**Figure 3.**
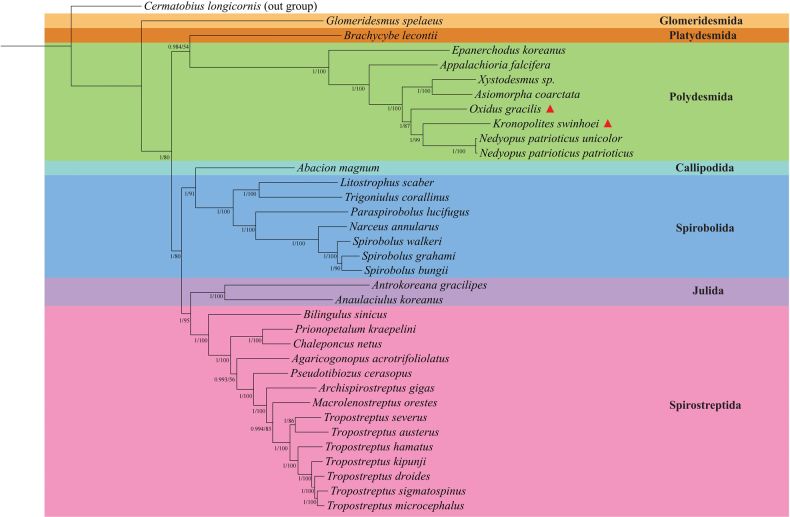
A phylogenetic tree was reconstructed from nucleotide sequences of 13 PCGs across 34 Diplopoda species, with *C. longicornis* designated as the outgroup. The tree was inferred using both Bayesian inference (BI) and maximum likelihood (ML) methods. Branch support is indicated as posterior probabilities (PP) for BI and bootstrap values (BS) for ML, presented in PP/BS format. Species newly sequenced in this study are marked with red triangles.

This study incorporated 34 diplopod species with reliably annotated mitogenomes from 15 families, using the chilopod *C. longicornis* as an outgroup to reconstruct the phylogenetic tree (Fig. 3). Both ML and BI analyses of the 13 PCGs produced identical topologies, with notably supported nodal values across clades. The resulting phylogeny strongly supports taxonomic relationships within the class Diplopoda. Current taxonomy places Julida, Spirostreptida, and Spirobolida within the superorder Juliformia, though their internal relationships remain contentious (Zhang et al. 2024c). In our phylogenetic analysis, Spirostreptida and Julida formed a distinct cluster, consistent with previous mitogenome studies that support a sister-group relationship between these two orders (Zuo et al. 2022; Zhang et al. 2024c; Li et al. 2025). In addition, morphological data suggest a sister-group relationship between Spirobolida and Julida (Blanke and Wesener 2014).

A key node uniting Platydesmida and Polydesmida received strong Bayesian posterior probability (PP = 0.984) but low bootstrap support (BS = 54%). As the ASDSF in the BI analysis was below 0.01, the PP value was considered more reliable for this clade (Alfaro et al. 2003). Within Polydesmida, representatives of the families Xystodesmidae and Paradoxosomatidae formed a highly supported clade (PP = 1, BS = 100%), consistent with previously reported topological structures (Zuo et al. 2022; Zhang et al. 2024b; Li et al. 2025). Although *X.* sp. and *Appalachioria falcifera* belong to the family Xystodesmidae, *X.* sp. clustered with *A. coarctata*, which belongs to Paradoxosomatidae, indicating that a precise phylogenetic placement of *X.* sp. warrants further thorough investigation. Within the Paradoxosomatidae clade, *O. gracilis*, *K. swinhoei*, and *N. patrioticus* formed a distinct group that was clearly separated from the cluster containing *X.* sp. and *A. falcifera*. Furthermore, *K. swinhoei* and *N. patrioticus* were placed in a well-supported clade (PP = 1, BS = 99%), whereas *O. gracilis* and *K. swinhoei* occupied separate, less-supported clades, providing robust molecular evidence for their taxonomic delineation. These results confirm that integrating molecular and morphological data yields more accurate classification.

Overall, the phylogenetic analyses shed light on millipede evolutionary history, biodiversity, and arthropod phylogeny. However, it is important to note that the dataset was limited to 34 species. Expanding taxonomic sampling in future sequencing efforts will be essential to resolve persisting disputes and clarify higher-level phylogenetic relationships among millipede.

## Conclusion

This study sequenced the complete mitogenomes of two species from the family Paradoxosomatidae. The overall mitogenomic architecture was highly conserved between *O. gracilis* and *K. swinhoei*, a pattern also observed in other members of Paradoxosomatidae. Notably, all genes were located on the N-strand, suggesting this may be a molecular synapomorphy for order Polydesmida. PCGs also exhibited a conserved arrangement, further supporting the notion of a characteristic genomic signature for the family Paradoxosomatidae. Two structurally conserved tRNA clusters were identified, and the rRNAs were separated by tRNA-Val. The D-loop region was located between tRNA-Ser2 and rrnS in both species. Consistent with other invertebrate mitogenomes, all genomic features displayed pronounced AT bias. Phylogenetic analyses based on these data offer new insights into millipede evolutionary history. However, the limited taxonomic sampling (34 species) constrains broader inferences. Expanding both taxon sampling and genomic data in future studies will be essential for resolving higher-level phylogenetic relationships within Diplopoda.

## Additional information

### Use of AI

No use of AI was reported.

### Author ORCIDs

Ming Gao https://orcid.org/0009-0007-5302-9287

Yingzhu Li https://orcid.org/0009-0004-9318-2663

Hongyi Liu https://orcid.org/0000-0003-2081-5779

### Data availability

All mitochondrial genome sequences produced in this study have been submitted to the NCBI database under the accession numbers PX619173 (*Kronopolites swinhoei*) and PX603245 (*Oxidus gracilis*).
